# Electrospun Zein/PCL Fibrous Matrices Release Tetracycline in a Controlled Manner, Killing ***Staphylococcus aureus*** Both in Biofilms and ***Ex Vivo*** on Pig Skin, and are Compatible with Human Skin Cells

**DOI:** 10.1007/s11095-015-1782-3

**Published:** 2015-09-03

**Authors:** Nour Alhusein, Ian S. Blagbrough, Michael L. Beeton, Albert Bolhuis, Paul A. De Bank

**Affiliations:** Department of Pharmacy and Pharmacology, University of Bath, Bath, BA2 7AY UK; Department of Biomedical Sciences, Cardiff Metropolitan University, Western Avenue, Cardiff, CF5 2YB UK

**Keywords:** biofilm, controlled drug delivery, electrospinning, nanofibre, pig skin

## Abstract

**Purpose:**

To investigate the destruction of clinically-relevant bacteria within biofilms via the sustained release of the antibiotic tetracycline from zein-based electrospun polymeric fibrous matrices and to demonstrate the compatibility of such wound dressing matrices with human skin cells.

**Methods:**

Zein/PCL triple layered fibrous dressings with entrapped tetracycline were electrospun. The successful entrapment of tetracycline in these dressings was validated. The successful release of bioactive tetracycline, the destruction of preformed biofilms, and the viability of fibroblast (FEK4) cells were investigated.

**Results:**

The sustained release of tetracycline from these matrices led to the efficient destruction of preformed biofilms from *Staphylococcus aureus* MRSA252 *in vitro*, and of MRSA252 and ATCC 25923 bacteria in an *ex vivo* pig skin model using 1 × 1 cm square matrices containing tetracycline (30 μg). Human FEK4 cells grew normally in the presence of these matrices.

**Conclusions:**

The ability of the zein-based matrices to destroy bacteria within increasingly complex *in vitro* biofilm models was clearly established. An *ex vivo* pig skin assay showed that these matrices, with entrapped tetracycline, efficiently kill bacteria and this, combined with their compatibility with a human skin cell line suggest these matrices are well suited for applications in wound healing and infection control.

## Introduction

Electrospinning is an established technique for the fabrication of nanoscale fibres (1–3). It continues to be studied extensively due to its various advantages such as high surface-to-volume ratio, tuneable porosity, and ease of surface functionalization. Indeed, the resulting fibres are extremely useful for applications in tissue engineering, drug delivery, and wound dressings. This is an area with huge potential for controlled release research. As electrospun fibres mimic the extracellular matrix (ECM) of tissues in terms of scale and morphology, there is the potential for them to be used as scaffolds. Taken together with their physical and chemical properties, electrospun scaffolds are being evaluated in various cellular studies, in sustained drug delivery, and as potential wound dressings (1–3). The localized and controlled drug delivery that might be achieved from electrospun micro/nanofibres could be applied in new treatments for burns or biomedical applications related to chronic wounds (1–3).

Burns and chronic wounds, such as diabetic ulcers, are hard to heal and require prolonged treatment due to a number of clinical complications (4, 5). There is an increasing interest in the topical application of antimicrobials to overcome the problems associated with the low levels of antibiotic in the granulating tissue (5). In future wound treatments, there may also be a desire to leave dressings on for extended periods, as this will minimise damage to newly formed tissue. However, current application of topical antimicrobials requires daily or twice-daily changes of the dressing, leading to patient discomfort as well as being time consuming and costly. There is therefore a considerable interest in dressings that allow the controlled release of antibiotics (6).

Biofilms are prevalent in nature and appear to be associated with the majority of infections, e.g. wound infections, catheter-linked infections, endocarditis, dental caries, and cystic fibrosis (7). Microbial communities infect chronic wounds and they often involve biofilms rather than planktonic cells (8–10). A particular problem of bacteria within biofilms is that they are significantly more resistant to antibiotics compared to their free-floating planktonic counterparts (11). Indeed, as a result of this increased resistance of biofilms to treatment by antibacterial agents, it is important to test the controlled release of an antibiotic matrix against bacterial biofilms. In wounds that require antibiotic treatment, localized antibiotic delivery systems may overcome the problems associated with the low antibiotic levels in the granulating tissue (5). We have recently developed formulations in which tetracycline (Tet) hydrochloride has been successfully incorporated in multi-layered electrospun micro/nanofibre matrices of zein and poly-ε-caprolactone (PCL) (12). We now report assays designed to test antibacterial activity achieved in a series of models of selected wound-associated biofilms of increasing complexity.

Alpha-zein is a corn (maize) protein containing a high percentage of non-polar amino acids with the ability to form aggregates and entrap solutes, such as drugs and amino acids. However, there are only a few papers, and those all recently published (12–16), reporting the incorporation of antibiotics within electrospun zein. Antimicrobial (chitosan) electrospun zein fibre structures provide a new strong antimicrobial ultrathin-structured system (13). Also, in order to develop biocompatible nanofibrous membranes for wound healing, the coelectrospinning of two proteins, zein and collagen, in aqueous acetic acid solution, was investigated where the combination with zein improved the electrospinning of collagen. The drug berberine was then incorporated *in situ* into the electrospun nanofibrous membrane, with little effect on fibre morphology and cell viability, in order to investigate its controlled release and antibacterial activity. Wound healing by these berberine releasing nanofibre membranes was examined *in vivo* using female Sprague Dawley rats and histology (14).

Luzardo-Alvarez and co-workers have used NMR spectroscopy to detect binding interactions and measure affinity between zein and three different drugs: indomethacin, and the antibiotics amoxicillin and Tet. Such protein-drug interactions show that zein is promising for the rational design of drug delivery vehicles (15). Luzardo-Alvarez *et al.* have also reported that treatment with Tet antibiotics within the periodontal pocket against *Staphylococcus aureus* bacterial infections represents a useful and adjunctive tool to conventional therapy for healing and teeth preservation. Thus, a two-polymer system of zein and PLGA has been developed as a biodegradable implant (16). Sustained release of Tet was obtained, and the proportion of zein in the inserts had a significant impact on the antibiotic release. Indeed, an effective release of Tet from the inserts against *S. aureus* achieved over 30 days of controlled delivery, and hence this may be suitable for the intra-pocket delivery of antimicrobial agents in the treatment of periodontitis (16).

In this paper, we report the incorporation of Tet in electrospun micro/nanofibre zein/PCL triple layers (3L) and its controlled release from these matrices (12). We show excellent antibiotic activity resulting in the destruction of clinically-relevant different *S. aureus* bacterial strains that are efficient biofilm formers, including activity against MRSA252, a representative of a lineage (EMRSA-16) that is endemic in UK hospitals (17). In particular, we investigate the biological activity of sustained release Tet in an *ex vivo* pig skin model relevant for wound dressing research and, for the first time, we report on the compatibility of such zein/PCL wound dressing matrices with human fibroblast (FEK4) skin cells.

## Materials and Methods

### Materials

Zein was purchased from Acros Organics. Poly-ε-caprolactone (PCL) (Mn 70 000–90 000), tetracycline (Tet) hydrochloride, other antibiotics and other chemicals, solvents, and membrane filters for microscopy (polycarbonate discs, pore size 0.2 μm, diameter 13 mm) were purchased from Sigma-Aldrich. Müller-Hinton (MH) agar, MH broth, Tryptone Soya broth (TSB), and antimicrobial tetracycline hydrochloride susceptibility test discs were purchased from Oxoid. Luria-Bertani (LB) broth, Costar polystyrene 96-well plates (flat bottom, volume 0.36 mL, well diameter 7 mm), and gene frames (1 × 1 cm) were purchased from Fisher. Crystal violet 1% was purchased from PRO-LAB Diagnostics. Filter discs were cut from Whatman 3MM Chr cellulose chromatography paper (0.34 mm thickness). The bacterial strains used in this study were *S. aureus* MRSA252 (17) and *S. aureus* ATCC 25923; these strains were maintained on Tryptone Soy Agar (Oxoid) plates.

### Preparation and Characterisation of Electrospun Matrices of Zein or Zein/PCL

Triple-layered (3L) matrices were prepared as recently reported (12). Zein solution was prepared at 30% (*w*/*v*) in a 1:1 (*v*/*v*) mixture of 2,2,2-trifluoroethanol (TFE):dichloromethane (DCM). Tet was dissolved in TFE at 5% (*w*/*w*) of the weight of zein. For blended matrices of zein and PCL, zein was dissolved at 20% and PCL at 10% in 1:1 (*v*/*v*) TFE:DCM, resulting in solutions with a total polymer concentration of 30% (*w*/*v*) with Tet (dissolved in TFE) again incorporated at 5% of the weight of the polymer. The polymer solution was loaded into a syringe and electrospun at 18 kV and a flow rate of 0.75 mL/h, with a distance between the tip of the needle and the collector of 13 cm. The flow rate was controlled by a syringe infusion pump (Cole Parmer, 230 VAC). The collector was constructed of two parallel metal electrodes covered with aluminium foil. 3L matrices consist of outer layers free of drug and an inner layer with Tet 5% of the weight of the polymer. To make these matrices, each polymer solution was electrospun using a fixed volume for each layer (1 mL for the outer layers and 0.5 mL for the inner layer) in a layer-by-layer manner. Two matrices were produced: triple-layered zein with Tet in the middle layer (zein 3L) and triple-layered zein/PCL with Tet in the middle layer (zein/PCL 3L). Three replicates of each formulation were fabricated. The viscosity of the electrospinning solutions was measured using a Bohlin high resolution C-VOR 200 Rheometer equipped with plate accessory using the spindle type CP4/40 maintained at 25°C. The shear rate was 50–100 Pa for the zein solution and 100–500 Pa for the zein/PCL solution. The surface morphology of electrospun matrices was observed by scanning electron microscopy (SEM) on matrices cut into small cm^2^ sized pieces (12).

### ***In Vitro*** Drug Release

The electrospun matrices were cut into 1.2 × 1.2 cm squares and, to minimise the effect of drug release from the edges, the samples were adhered to plastic coverslips using a gene frame to give an available release surface of 1 cm^2^. Samples were placed under sink conditions in plastic vials containing phosphate buffered saline (PBS, 5 mL, pH 7.4) and incubated at 37°C. At set time-points, the PBS was replaced and Tet release was determined in the sampled buffer by measuring its UV absorbance at λ = 360 nm against a standard curve. Cumulative Tet release was determined by comparing the mass released at each time point with the theoretical mass of Tet encapsulated in each sample. Triplicate samples were examined for each formulation and the experiment was performed three times with independently electrospun mats (12).

### MIC Planktonic Bacteria Assay

Minimum inhibitory concentration (MIC) values against planktonic bacterial cells were determined with a microdilution broth method using MH Broth as previously described (18).

### 96-Well Microtiter Plate (MTP) Biofilm Assay

The effect of antibiotics on biofilms of *S. aureus* MRSA252 was tested as we have recently described (19–21), with minor modifications. Briefly, these bacterial cells were cultured (15 h) in TSB containing 0.5% glucose and 3% NaCl (TSB-GN) and diluted 20-fold. The diluted bacterial suspension (200 μL/well) was dispensed into a polystyrene 96-well plate, and the plates were incubated for 24 h at 37°C on a 3-dimensional plate rotator (40 rpm). The cell suspension was removed and the biofilms were washed carefully with sterile PBS (200 μL).

The effectiveness across a panel of antibiotics was then tested on preformed biofilms on the same plate, therefore on the same day and under the same conditions. To test the antibiotics, 200 μL fresh TSB-GN (control wells) or TSB-GN containing the appropriate concentration of antibiotic (50 μg/mL) was added to preformed (24 h) biofilms, and the plates were then incubated for a further 24 h. After this, non-adherent cells were removed, and the biofilms were washed with sterile PBS (3 × 200 μL/well). The plates were dried (1 h at 20°C) and biofilms were then stained with crystal violet solution (1% *w*/*v*). After 15 min, the excess of crystal violet was removed, plates were washed briefly with water (4 × 250 mL), and the crystal violet was dissolved in aqueous acetic acid (30% *v*/*v* in distilled water). The absorbance, representative of the amount of biofilm remaining after treatment, was measured at λ = 595 nm (A_595_) using a FLUOstar Omega spectrophotometer (BMG LABTECH, UK).

To test the effectiveness of Tet loaded electrospun matrices, fresh TSB-GN (200 μL) was added to preformed (24 h) biofilms of *S. aureus* MRSA252, and then matrices (6 mm diameter) or Tet solution were added to each well in 7 groups, with 3 replicates: matrices containing 30 μg Tet (zein 3L, zein/PCL 3L), matrices containing no Tet (single layer zein no Tet, zein/PCL no Tet), Tet solution (30 μg/well), a negative control (media+Tet solution 30 μg/well, in wells without biofilm), and a positive control (wells with biofilm, only fresh medium added). Plates were incubated again for 24 h at 37°C. The next day, the same discs were transferred to newly preformed biofilms, incubated for 24 h at 37°C, and this was repeated a third time. After each treatment, the bacterial suspension was removed and the biofilms were washed, stained, and analysed as above (19–21).

### Colony Biofilm Model (CBM)

The effect of Tet loaded electrospun matrices on biofilms was tested in the CBM as recently described (20, 22), with minor modifications. *S. aureus* MRSA252 was cultured (15 h) in TSB-GN, and diluted to an OD at 600 nm of 0.4–0.6. Three sterile 13 mm polycarbonate discs were placed on the surface of TSB agar plates and aliquots (50 μL) from the diluted bacterial suspension were spotted on to each disc. Inoculated discs were incubated for 72 h at 37°C to allow formation of the biofilm. The polycarbonate discs were carefully transferred to new agar plates with sterile forceps on a daily basis. On the fourth day, the discs were covered with zein 3L or zein/PCL 3L (13 mm diameter). The third polycarbonate disc was left without any treatment. Before covering the discs with the 3L matrices, the surface of the biofilms was wetted with TSB (10 μL) and, after coverage, another 20 μL of broth was applied on top of the matrices. The plates were then incubated again at 37°C for 24 h. Each disc was then transferred to a tube containing TSB (5 mL) and kept cool on ice during the experiment. The tubes were vortexed extensively (~5 min) in order to disrupt mechanically the biofilms and detach the bacteria from the discs. Suspended cells were then serially diluted to 10^−7^ in broth, and aliquots (10 μL) of 10^−4^, 10^−5^, 10^−6^, and 10^−7^ dilutions were spotted on TSB agar plates, to determine the colony forming units (CFU)/mL using the Miles-Misra method (23). The plates were incubated at 37°C for 24 h and the numbers of CFU were counted. The number of CFU/disc was determined using the following formula:

CFU/disc = CFU counted × dilution factor × 100 × 5; this was then normalised by the number of CFU/biofilm disc.

### ***Ex Vivo*** Pig Skin Infection Model

The routine preparation of the pig skin followed this procedure. Thawed pig skin (ex-abattoir, stored for weeks frozen) was epilated with a dry razor, stripped with a tape 10-times to remove the upper dead layers, and finally cut into 1 × 1 cm^2^ pieces. The pieces were sterilised firstly by immersing in 70% ethanol for 20 min, dried for 20 min in a biosafety cabinet, followed by soaking in a solution of antibiotics for 16 h (kanamycin sulfate 20 μg/mL and ampicillin 50 μg/mL). On the day of the experiment, a cut 0.9 cm long was created along the skin to reach the epidermal layer using a sharp knife. The cut pig skin squares were then placed on TSB agar plates prepared with antibiotics (kanamycin sulfate 20 μg/mL and ampicillin 50 μg/mL).

*S. aureus* MRSA252 or ATCC 25923 were cultured (15 h) in TSB-GN. Inoculation was with an aliquot (20 μL) of bacterial suspension applied to the epidermal side of the skin and spread uniformly. The inoculated skin pieces were incubated for 5 days at 37°C in a humidified chamber. Every day the pieces were transferred to a new agar plate. Four pig skin pieces were used in each repeat. On day 5, the pig skin pieces were covered with zein 3L, zein/PCL 3L (1 cm^2^), or a filter paper loaded with 30 μg Tet. A fourth pig skin piece was left untreated. Before covering the pig skin pieces with the 3L matrices, the surface of the biofilms was wetted with TSB-GN (10 μL) and, after coverage, another 20 μL of broth was applied on top of the matrices. The plates were then incubated again at 37°C for 24 h. Each skin piece was then transferred to a tube containing MH broth (5 mL) and kept cool on ice during the experiment. The tubes were vortexed extensively (~5 min) in order to detach mechanically the bacteria from the skin. Suspended cells were then serially diluted to 10^−7^ in broth, and aliquots (10 μL) of 10^−4^, 10^−5^, 10^−6^, and 10^−7^ dilutions were spotted on TSB-GN agar plates, to determine the colony forming units (CFU)/mL using the Miles-Misra method (23). The plates were incubated at 37°C for 24 h and the numbers of CFU were counted. The number of CFU/skin piece was determined using the above formula; this was then normalised by the number of CFU/untreated skin piece.

### Cell Viability Test (MTS Assay)

Single layer zein and zein/PCL electrospun matrices containing 5% Tet were punched into 6 mm diameter discs and fixed to the bottom of the wells of 96-well plates using pieces of gene frame. FEK4 cells are passage-dependent human primary foreskin fibroblasts. They were routinely cultured in Earle’s modified minimum essential medium supplemented with 10% fetal calf serum (heat-inactivated at 56°C for 45 min before use) and 50 IU/mL of penicillin and streptomycin, and maintained at 37°C in a humidified incubator with 5% CO_2_. FEK4 cells between passage 11 and 17 were seeded in each well at 750 cells/100 μL and incubated for 3 days before the relative cell number was assessed using the MTS assay. On the day of the assay, MTS reagent (20 μL) was added to each well and incubated for another 4 h at 37°C. After incubation, culture medium (50 μL) was then transferred from each well to a new 96-well plate and the absorbance of the solutions measured at 490 nm (*n* = 3, in triplicate). Tet solution alone and cells seeded directly on the tissue culture plastic wells were used as controls.

Student’s t-tests were performed using Microsoft Excel to determine any statistically significant differences between the formulations and the commercially available Tet filter discs; *p* < 0.05 is considered to be significant.

## Results and Discussion

### 96-Well Microtiter Plate (MTP) Assay of Antibiotic Activity in Destroying MRSA Biofilms

A now well-established consequence of the formation and maturation of microbial biofilms is increased resistance to antibiotics and hence their failure in therapy (24). In part, this occurs by preventing access of the antibiotics to their sites of action, together with other mechanisms still not completely understood (24). We have tested a range of clinically important antibiotics against preformed MRSA252 biofilms using the Microtitre Plate (MTP) assay model.

**Fig. 1:**
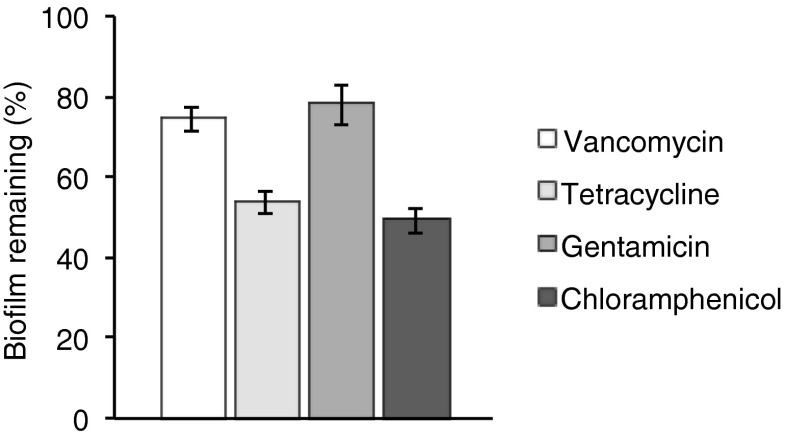
The activity of clinically used antibiotics (200 μL containing 50 μg/mL, for 24 h) against preformed (24 h) MRSA252 biofilms (normalised relative to crystal violet assayed biofilm formation in the absence of any antibiotic) tested using the MTP model showing the efficiency of biofilm removal. The *error bars* represent the standard deviation (*n* = 3, in triplicate).

The data show (Fig. 1) that vancomycin, the glycopeptide often named as the antibiotic of last resort for certain multi-drug resistant Gram-positive infections, and gentamicin are only weakly active against this *S. aureus* strain once biofilms have formed. However, tetracycline (Tet), used for the treatment of skin infections, and chloramphenicol, less widely used except in over the counter (OTC) eye-drops against conjunctivitis, were active removing ~50% of the biofilms (Fig. 1). In addition, we have also assayed the MIC values of these antibiotics against planktonic MRSA252 bacterial cells. The MIC values are: gentamicin 1 μg/mL; Tet 0.25 μg/mL; vancomycin 0.25 μg/mL; chloramphenicol 16 μg/mL (18). These MIC values are considerably (200-fold for Tet) lower than the concentrations used (Fig. 1) in destroying MRSA biofilms. Gentamicin is known to be less active against intracellular bacteria (25, 26), but that is not relevant in the MTP assay where the bacteria are adhered to polystyrene wells. The activity of these antibiotics against planktonic cells is significantly higher, and the lower activity is due to the biofilm state of the bacteria. Lack of biological activity against biofilms is due to a number of factors, including penetration of the antibiotic into the biofilm, and the low rates of cell growth in a biofilm as many cells in a biofilm are in a semi-dormant state (27).

With regard to potential wound dressings, we have therefore focussed our research on the sustained and controlled release of Tet in *in vitro* and *ex vivo* models of increasing complexity with bacterial targets where biofilms are known to be a clinical problem. Interestingly, Tet is normally considered bacteriostatic, yet at the concentrations used here it results in the removal of the biofilms. However, it should be noted that at concentrations > 0.3 μg/mL Tet was shown to be bactericidal for *S. aureus* (28). The concentrations used here are significantly above that value, and thus it is likely that Tet kills cells in the biofilms, albeit rather inefficiently. We have therefore designed and used a variety of assays in order to evaluate the antibacterial efficacy of electrospun Tet-loaded matrices including: a 96-well MTP biofilm assay, a colony biofilm model, and an *ex vivo* pig skin model.

### Characterization of Electrospun Matrices of Zein or Zein/PCL

The diameter of electrospun zein fibres (in triple layers, 3L) was 0.99 ± 0.36 μm (12). Electrospun zein/PCL fibres were thicker than zein fibres and had more variability, with diameter of 1.51 ± 0.65 μm for zein/PCL (20:10) 3L. This can be explained, in part, by the difference in viscosities displayed by the polymeric solutions. The zein solution viscosity was 0.186 ± 0.012 Pa.s, whereas the zein/PCL solution viscosity was much higher at 1.84 ± 0.24 Pa.s. The increase in viscosity may indicate a greater polymer chain entanglement in the solution. Applying the same voltage to the more viscous zein/PCL solution led to less jet stretching during the electrospinning process, resulting in a larger fibre diameter.

### Tet Release from Triple-Layered Matrices

Prior to release studies, the encapsulation efficiency of each matrix formulation was determined with mats cut into small discs (~6 mm diameter), weighed and then dissolved in methanol:DCM (1:1 *v*/*v*, 10 mL). From the UV absorbance (λ = 360 nm, subtracting for zein absorbance at this wavelength), the amount of Tet HCl in the fibres was then calculated using a Tet HCl calibration curve and subsequently compared to the theoretical value (5%). Within experimental error, encapsulation was quantitative for all but the zein/PCL (20:10) 3L samples which had an encapsulation efficiency of 71 ± 11% (12).

**Fig. 2:**
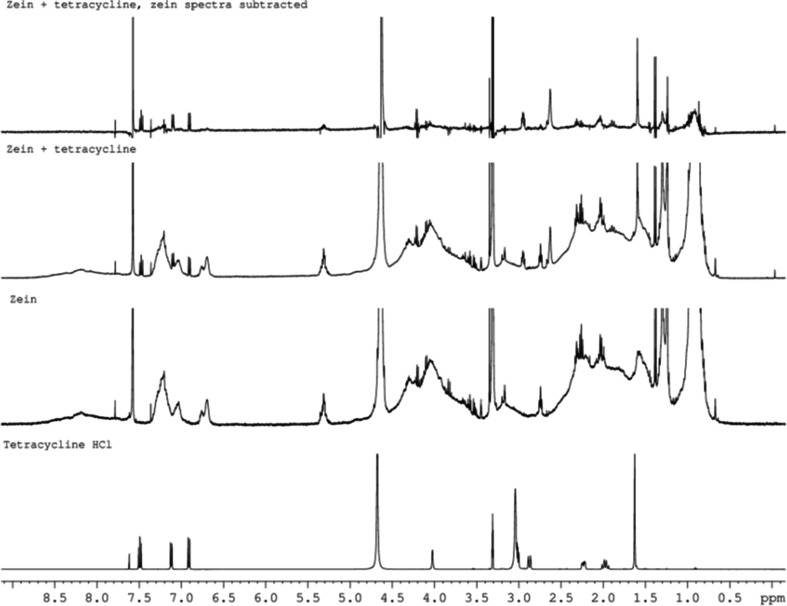
^1^H NMR spectra (CDCl_3_) of (in ascending order): Tet, Tet-free electrospun zein, electrospun zein matrix containing Tet, and electrospun zein containing Tet but with the zein spectral data subtracted.

It is well known that light, temperature, moisture, and duration of storage influence the stability of Tet leading to a decrease in its microbiological activity and an increase in its toxicity (29). The physical and chemical stability of Tet in these electrospun zein matrices has therefore been examined in detail (12). Following our recent precedent with electrospun PCL matrices (29), we have applied ^1^H NMR spectroscopy (Fig. 2) showing that the chemical integrity of loaded Tet HCl was maintained after the electrospinning process as all the signals of Tet HCl could be observed, comparable with the literature data (29). We have also demonstrated spectroscopic (UV) and spectrometric (MS) stability of electrospun Tet, and employed Raman microscopy to demonstrate the even distribution of Tet in the electrospun fibres (12). Significantly, we also show, *vide infra*, that the electrospun Tet is still biologically active, working equally effectively in comparison with commercial Tet.

**Fig. 3:**
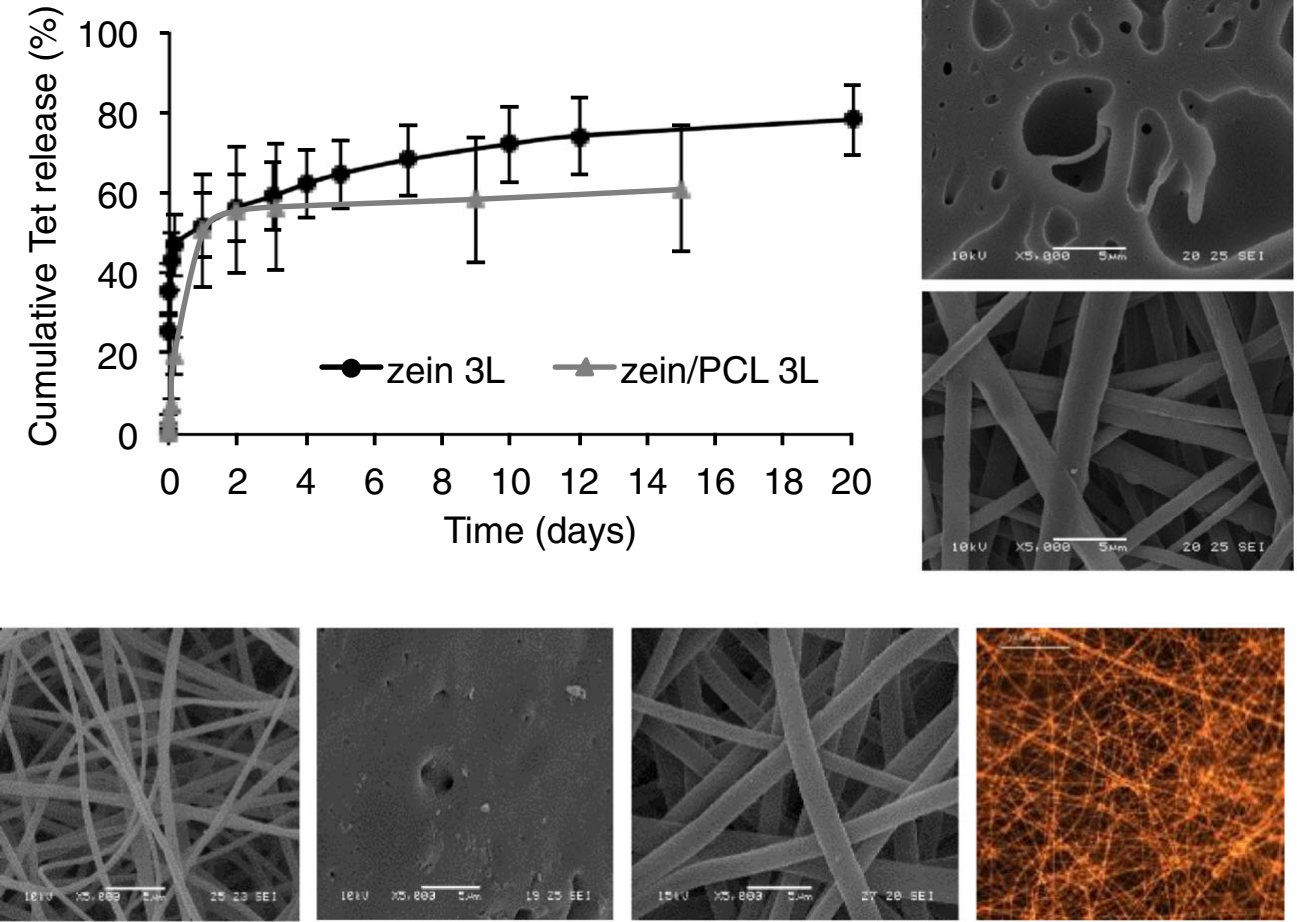
The Tet release profile from the electrospun triple-layered electrospun matrices zein 3L and zein/PCL 3L with the Tet payload (5%) encapsulated in the middle layer only. The *error bars* represent the standard deviation (*n* = 3, in triplicate). The insets on the right show representative SEM images of electrospun zein 3L (*upper*) and electrospun zein/PCL (20:10) 3L (*lower*) matrices after immersion in PBS at 37°C (12). Below are other representative images showing, from the *left*: electrospun zein 3L, electrospun zein 3L after immersion in PBS at 37°C, electrospun zein/PCL (20:10) after immersion in PBS at 37°C, and a fluorescence microscopy image of Tet HCl loaded zein.

In our previous *in vitro* study using electrospun zein (12), a fast release of Tet from electrospun triple-layered zein (3L) was observed within the first 3 h (47% of the encapsulated drug). We have also previously shown that zein matrices shrink in aqueous media and lose their fibrous structure becoming a film (12) (Fig. 3). In order to overcome this, we blended PCL with zein. This successfully maintained the fibrous structure on contact with water and stopped the shrinkage (12) (Fig. 3). In addition, zein/PCL 3L showed a more gradual release of Tet as only 19% was released within the first 3 h. However, both formulations liberated 50% of the encapsulated Tet after 24 h (Fig. 3). In the following days, zein 3L sustained the release of Tet up to 20 days in which a further 27% of encapsulated Tet was released. Whereas zein/PCL 3L released a further 10% of Tet until day 15 when the release plateaued. In the first few hours, zein/PCL 3L showed a better gradual release than zein 3L, however, zein/PCL 3L released Tet in the following days very slowly compared to zein 3L. It is possible that the addition of PCL to the formulation increased the hydrophobicity of the layered matrix and thus restricted the water access to the drug encapsulated within the polymeric blended fibres.

### Effect of Matrices on Biofilms Measured in the MTP Assay

**Fig. 4:**
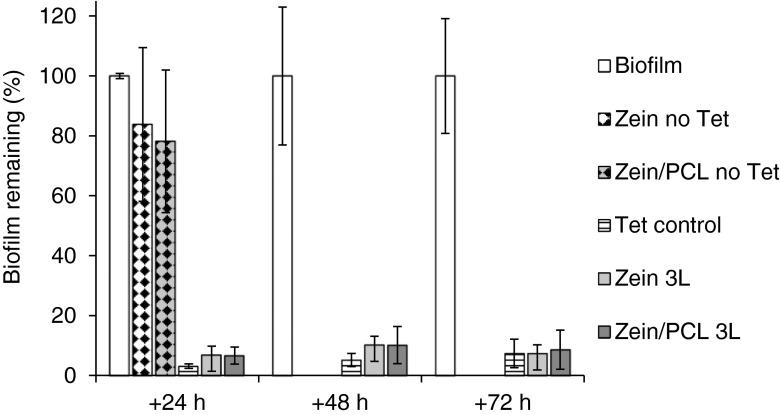
The activity of the electrospun matrices against preformed MRSA252 biofilms (normalised absorbance) tested using the MTP model showing the percentage of biofilm remaining after treatment, where the untreated biofilm acts as a control for normalisation. The *error bars* represent the standard deviation (*n* = 3, in triplicate).

The MTP assay has the advantage of being a simple, rapid, and inexpensive screen of anti-biofilm agents using crystal violet (19, 30). We modified the design of the assay in order to investigate the antibacterial effect of sustained Tet release and observed that 3L matrices significantly decreased the biomass of biofilms formed by *S. aureus* MRSA252, and remained bioactive when re-used on fresh biofilms (for three consecutive days; more than 90% decrease, *p* < 0.001; Fig. 4). Comparing 3L matrices to the Tet control results, Tet control decreased the absorbance more than the triple matrices did in the first 48 h (*p* < 0.05), while there was no significant difference after 72 h (*p* > 0.05). This is due to the fact that a significant proportion of the Tet load in the 3L matrices was encapsulated in the polymeric fibrous matrices and not rapidly released.

### Colony Biofilm Model (CBM)

**Fig. 5:**
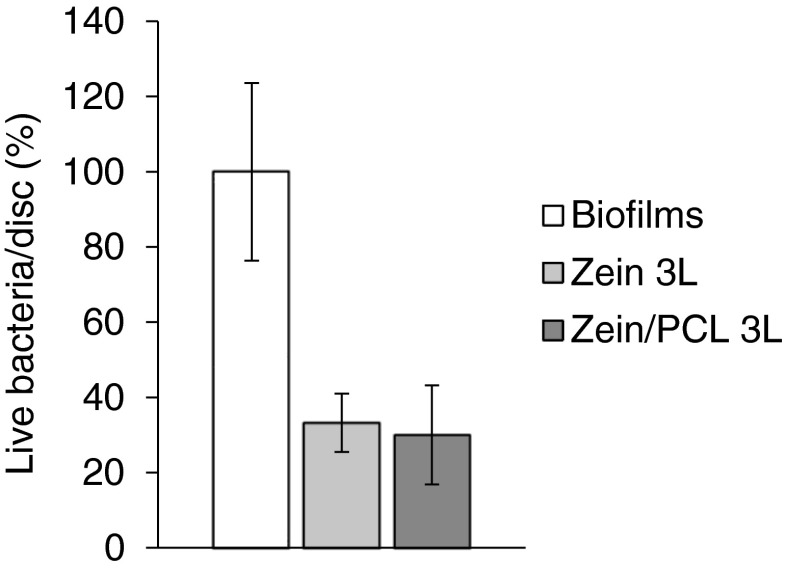
Effects of the Tet containing electrospun matrices on MRSA252 biofilms grown on polycarbonate discs tested using the CBM model expressed as normalised live bacteria/disc. The *error bars* represent the standard deviation (*n* = 3).

In this model, biofilms were grown on a polycarbonate membrane that sat on top of an agar plate in a system that mimics biofilms growing in a wound (20, 22). The low fluid shear and the proximity to an air interface provided by this model simulate the wound environment (31). Additionally, the nutrient flow is similar to that of biofilms in a wound, with carbon and nitrogen sources (usually from the host tissue *in vivo*) coming from the agar, and the oxygen diffusing into the biofilm from the air interface on the opposite side of the biofilm (20, 22, 31). Figure 5 shows the antibacterial effectiveness of the formulations. Compared with the control biofilms, the 3L matrices significantly reduced the number of living cells in the biofilms on the polycarbonate discs. The CFU/disc was reduced from 100% for untreated biofilms to ~35% for 3L matrices (*p* < 0.01). There was no significant difference in CFU/disc achieved with zein 3L compared to zein/PCL 3L (*p* > 0.05).

### ***Ex Vivo*** Pig Skin Infection Model

Pig skin and human skin share many physiological and anatomical similarities. For example, both pig and man have a thick epidermis (50 to 120 μm in human compared to 30 to 140 μm in pigs). They both enjoy well-developed rete-ridges, papillary bodies, and abundant subdermal adipose tissue. They also demonstrate a similar size, orientation, and distribution of blood vessels, adenexal structures, type of keratinous proteins, collagen, body hair and lipid composition of the stratum corneum (32). Here, an *ex vivo* pig skin model was used to evaluate the antimicrobial efficacy of the 3L electrospun matrices against two bacterial strains, MRSA252 and ATCC 25923.

**Fig. 6:**
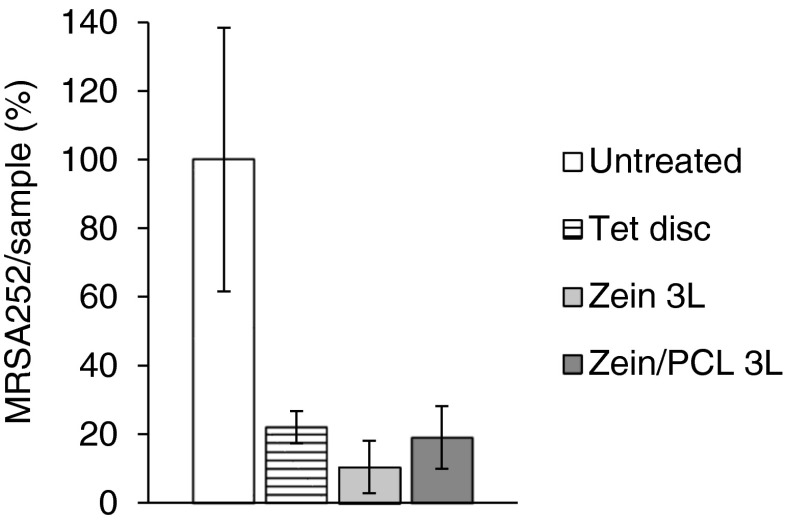
The antibacterial effects of Tet loaded formulations after 5 days of incubating MRSA252 on pig skin, 37°C. The formulations were added on the fifth day and left for 24 h (*n* = 3). The *error bars* represent the standard deviation.

Figure 6 shows the antibacterial effectiveness of the formulations against MRSA252 when grown on pig skin for 5 days *ex vivo*. Compared with the untreated pig skin samples (as a normalised control), the Tet loaded 3L matrices significantly reduced the number of living cells; the CFU/skin sample was reduced from 100% for untreated samples to 22% for commercial Tet impregnated filter discs, 19% for zein/PCL 3L matrices, and 10% for zein 3L matrices (all *p* < 0.01). We did note, however, that *S. aureus* MRSA252 did not grow particularly well on pig skin, and for that reason another *S. aureus* strain, but a meticillin sensitive (MSSA) strain (ATCC 25923) was also used. This strain grew significantly better on pig skin and, similar to MRSA252, treating the infected pig skin with Tet loaded zein/PCL 3L matrices also led to a significant reduction in the number of CFU of ATCC 25923 (from 100 ± 39% for the untreated sample to 27 ± 4% for the treated sample, *p* < 0.01). Thus, these Tet loaded 3L matrices efficiently killed both *S. aureus* MRSA252 and MSSA ATCC 25923 grown for 5 days on pig skin *ex vivo* and then treated with the matrix formulation for 24 h.

### Cell Viability Test (MTS Assay)

**Fig. 7:**
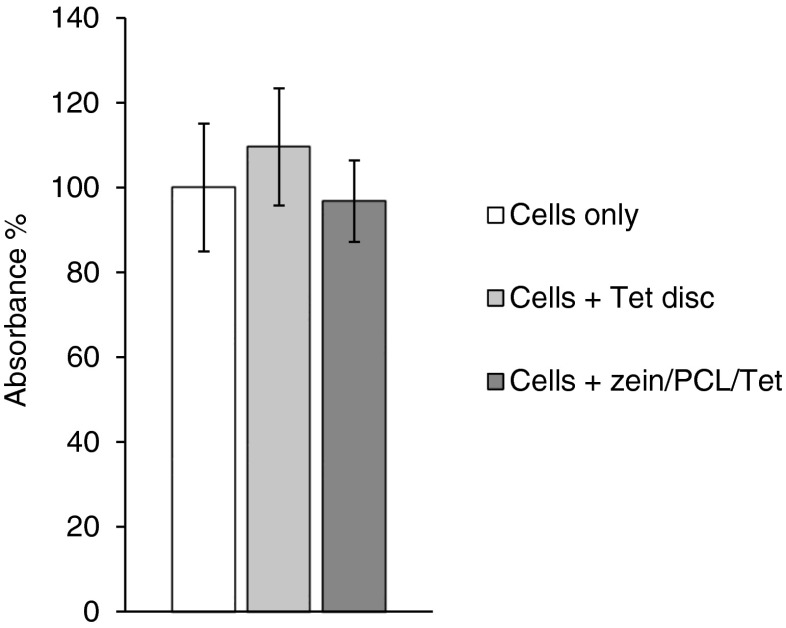
The MTS cell metabolism assay (normalised absorbance) demonstrates the biocompatibility of zein/PCL+Tet, 72 h after seeding. The *error bars* represent the standard deviation (*n* = 3, in triplicate).

Having established in three different models of bacterial biofilm formation that these electrospun fibrous 3L matrices are releasing bioactive Tet in a sustained manner, we wanted to show that this formulation is not toxic to human skin cells. The 3-(4,5-dimethylthiazol-2-yl)-5-(3-carboxymethoxyphenyl)-2-(4-sulfophenyl)-2H-tetrazolium (MTS) assay was therefore used to demonstrate that there was no adverse effect on the growth of human primary skin fibroblast (FEK4) cells seeded on zein/PCL+Tet electrospun fibrous matrices (single layer). Commercially available Tet impregnated filter discs were used as a control to investigate whether Tet is toxic to these fibroblast cells or not. Figure 7 shows that the metabolic activity of the cells 72 h after seeding on the zein/PCL+Tet electrospun mats was similar to that of cells seeded on the tissue culture plastic with or without the commercial Tet filter discs (*p* > 0.05), demonstrating that the zein/PCL blend, as a biomaterial, supported fibroblast adhesion and growth, and that the release of Tet from this formulation had no detrimental effect. This biocompatibility of soluble Tet with these cells was confirmed by the observation that commercially available Tet filter discs did not affect the growth of FEK4 cells on tissue culture plastic (Fig. 7). These results, demonstrating the cellular compatibility of zein, are similar to the results within a recent study examining the growth on murine fibroblasts on zein nanofibres containing different concentrations of curcumin (33). Therefore, these zein/PCL electrospun matrices provide an attractive structure for the attachment and growth of fibroblasts as cell culture surfaces and so they present a suitable candidate for potential further applications in drug delivery systems. Indeed, a related application on the release of the antibiotic amoxicillin from electrospun fibrous wound dressing patches has recently been reported in this *Journal* (34).

## Conclusions

The potential and feasibility of a blend of zein and PCL as a drug delivery vehicle using designed and engineered electrospun matrices has been investigated. As electrospun Tet encapsulated zein fibres (i.e. zein without PCL) shrank significantly in aqueous media, Tet encapsulated micro/nanofibre zein/PCL 3L electrospun matrices were prepared, where blending with PCL stopped the shrinkage (12). Tet release was controlled for over 10 days demonstrating a potential for application in wound treatments e.g. as dressings or even as implants. In this paper, we demonstrate that this sustained released Tet showed excellent antibiotic activity in destroying preformed biofilms from *S. aureus* MRSA252 in models resembling the situation found in wounds. In addition, applying these Tet loaded matrices, for the first time, to *ex vivo* pig skin models, growing either MRSA252 or ATCC 25923, significantly reduced the bioburden of these clinically relevant bacteria. These zein/PCL electrospun matrices were also shown to be compatible with human fibroblast FEK4 skin cells. Taken together, the clearly established ability of these Tet loaded zein-based matrices to destroy bacteria within increasingly complex *in vitro* and *ex vivo* biofilm models and their biocompatibility with human skin cells lead to the conclusion that these matrices could be well suited for applications in wound healing and infection control.

## Abbreviations

3L: Triple-layered
CBM: Colony Biofilm Model
DCM: Dichloromethane
MIC: Minimum inhibitory concentration
MTP: 96-Well Microtiter Plate
OD: Optical density
TFE: 2,2,2-Trifluoroethanol
TSB: Tryptone Soya broth
TSB-GN: Tryptone Soya broth containing 0.5% glucose and 3% NaCl
